# Dental caries in primary and permanent teeth in children’s worldwide, 1995 to 2019: a systematic review and meta-analysis

**DOI:** 10.1186/s13005-020-00237-z

**Published:** 2020-10-06

**Authors:** Mohsen Kazeminia, Alireza Abdi, Shamarina Shohaimi, Rostam Jalali, Aliakbar Vaisi-Raygani, Nader Salari, Masoud Mohammadi

**Affiliations:** 1 Department of Nursing, School of Nursing and Midwifery, Kermanshah University of Medical Sciences, Kermanshah, Iran; 2 Department of Biology, Faculty of Science, University Putra Malaysia, Serdang, Selangor Malaysia; 3 Department of Biostatistics, School of Health, Kermanshah University of Medical Sciences, Kermanshah, Iran

**Keywords:** Caries, Tooth, Primary and permanent, Prevalence, Meta-analysis

## Abstract

**Background:**

Early childhood caries (ECC) is a type of dental caries in the teeth of infants and children that is represented as one of the most prevalent dental problems in this period. Various studies have reported different types of prevalence of dental caries in primary and permanent teeth in children worldwide. However, there has been no comprehensive study to summarize the results of these studies in general, so this study aimed to determine the prevalence of dental caries in primary and permanent teeth in children in different continents of the world during a systematic review and meta-analysis.

**Methods:**

In this review study, articles were extracted by searching in the national and international databases of SID, MagIran, IranMedex, IranDoc, Cochrane, Embase, ScienceDirect, Scopus, PubMed, and Web of Science (ISI) between 1995 and December 2019. Random effects model was used for analysis and heterogeneity of studies was evaluated by using the I^2^ index. Data were analyzed by using the Comprehensive Meta-Analysis (Version 2) software.

**Findings:**

In this study, a total of 164 articles (81 articles on the prevalence of dental caries in primary teeth and 83 articles on the prevalence of dental caries in permanent teeth) were entered the meta-analysis. The prevalence of dental caries in primary teeth in children in the world with a sample size of 80,405 was 46.2% (95% CI: 41.6–50.8%), and the prevalence of dental caries in permanent teeth in children in the world with a sample size of 1,454,871 was 53.8% (95% CI: 50–57.5%). Regarding the heterogeneity on the basis of meta-regression analysis, there was a significant difference in the prevalence of dental caries in primary and permanent teeth in children in different continents of the world. With increasing the sample size and the year of study, dental caries in primary teeth increased and in permanent teeth decreased.

**Conclusion:**

The results of this study showed that the prevalence of primary and permanent dental caries in children in the world was found to be high. Therefore, appropriate strategies should be implemented to improve the aforementioned situation and to troubleshoot and monitor at all levels by providing feedback to hospitals.

## Background

Early childhood caries (ECC) is a type of dental caries in the teeth of infants and children that is represented as one of the most prevalent dental problems in this period [1] which can lead to pain, infection, interference with eating, increased risk of new dental caries in primary and permanent teeth, and, ultimately, worse effects on the eruption of permanent teeth [2]. These manifestations can range from demineralization to loss of tooth structure or complete destruction of the crown, a process of dynamic and active decay characterized by various periods of destruction and repair [3].

According to the American Academy of Dentistry, early childhood caries (ECC) is defined as “the presence of 1 or more decayed (non-cavitated or cavitated lesions), missing (due to caries), or filled tooth surfaces in any primary tooth” in children [1]. Overall, 50% of children have one or more decayed primary teeth by the end of toddler age, but the importance of these teeth should not be overlooked, because, as has been said, healthy teeth in childhood have an important role in the eruption of healthy permanent teeth, healthy nutrition, and one’s aesthetic appearance [2, 3]. Factors such as malnutrition, genetic predisposition, poor health performance, specific eating habits, the presence of organisms affecting tooth decay such as streptococci, and fluoride and vitamin D deficiency, excessive sugar consumption and prolonged bottle-feeding, and other factors such as age, gender and place of residence of children are effective in causing tooth decay [4].

The World Health Organization (WHO) has represented the early childhood caries as a worldwide problem with a prevalence of between 60 and 90% [5]. According to the statistics provided by the European countries, 61% of children aged 6 to 12 years have at least one decayed tooth, and due to widespread dental caries in all social classes, this disease can impose a great financial burden on the society [6]. In Iran the mean decay-missing-filled (DMF) index of primary teeth in children aged 3 to 6 years was 1.7 and DMF index of permanent teeth was reported to be 0.2 in 6- to 9-year-old children, 0.9 to 1.5 in 12-year-old children, and 3.3 to 4.8 in 9-year-old children [7]. The decay-missing-filled (DMF) index is used as an appropriate measure for the detection of dental caries in the society in which 12-year-old children are considered as a target group [8].

Primary teeth begin to erupt in infants’ mouths at about 6 months of age, and are completed at age 3 to 5, including 10 teeth in the maxilla and 10 in the mandible to meet nutritional needs in infancy [6]. Since primary teeth are the basis of permanent teeth, on the one hand, and they have a high susceptibility to caries, on the other hand, these teeth are very important and maintaining their health is considered a serious health concern for children [3, 9].

Due to the influence of different factors on the prevalence of primary and permanent dental caries in children and lack of general statistics about this issue worldwide, we decided to review the studies in this area and to statistically analyze the results of these studies to compile a general statistics on the prevalence of dental caries in primary and permanent teeth in children in different continents of the world to open a window to more precise planning to reduce the complications of primary and permanent dental caries in children.

## Methods

In this systematic review and meta-analysis, the prevalence of dental caries in primary and permanent teeth in children was evaluated based on studies conducted between 1995 and December 2019. To this end, articles published in the national databases of SID, MagIran, IranMedex, and IranDoc, and in the international databases of Google scholar, Cochrane, Embase, ScienceDirect, Scopus, PubMed, and Web of Science (ISI) were searched by using Persian and English keywords such as Prevalence, Caries, Rampant caries, Milky tooth, Permanent tooth and Children.

### Selection of studies

Initially, all articles referring to the prevalence of dental caries in primary and permanent teeth in children in the world were collected by the researchers and accepted based on the inclusion and exclusion criteria. The inclusion criteria were observational (non-interventional) studies and their full text availability. For more information, the sources of the articles reviewed were also reviewed for access to other articles.

Exclusion criteria included irrelevant cases, case reports, interventional studies and other review, case-control, cohort, duplication of studies, unclear methodology, and full text unavailability. In order to reduce bias, the articles were searched independently by two researchers, and if they disagreed on a study, the article was reviewed by the group supervisor (blinded about the decision by the first two independent researchers’ decision). A total of 180 studies entered the third stage, quality assessment.

Duplicate publication and multiple publications from the same population will be removed using citation management, software EndNote (version X7, for Windows, Thomson Reuters).

### Quality assessment of studies

The quality of the articles was first evaluated on the basis of selected and related items of the 22- item STROBE checklist that could be evaluated in this study (study design, background and literature review, place and time of study, outcome, inclusion criteria, sample size, and statistical analysis) and also mentioned in the previous studies. Articles referring to 6 to 7 criteria were considered as high quality articles, articles that did not mention 2 items and more than 2 items from the seven items were considered as medium and low methodological quality articles, respectively [10]. In the present study, 164 articles with high quality and medium quality were entered the systematic review and meta-analysis, and 16 articles were of poor quality and were excluded.

### Data extraction

All final articles entered into the meta-analysis process were prepared to be extracted by a pre-prepared checklist. The checklist included article title, first author’s name, year of publication, place of study, sample size, mean age of sample, prevalence of dental caries in primary and permanent teeth.

### Statistical analysis

Since prevalence has binomial distribution, prevalence variance was calculated using the binomial distribution variance formula, and weighted mean was used to combine the prevalence rate of different studies. In order to evaluate the heterogeneity of the selected studies, I^2^ test was used (heterogeneity was divided into three classes of less than 25% (low heterogeneity), 25–75% (moderate heterogeneity) and more than 75% (high heterogeneity). Meta-regression analysis was used to investigate the relationship between the prevalence of dental caries with the sample size and the year of study. In order to evaluate the publication error with respect to the large sample size of studies entered the review, the Begg and Mazumdar test at the significant level of 0.1 and its corresponding Funnel plot were used. Sensitivity analysis was used to evaluate the effect of individual studies on the final result. Data were analyzed by using the Comprehensive Meta-Analysis (Version 2) software.

## Results

**Fig. 1:**
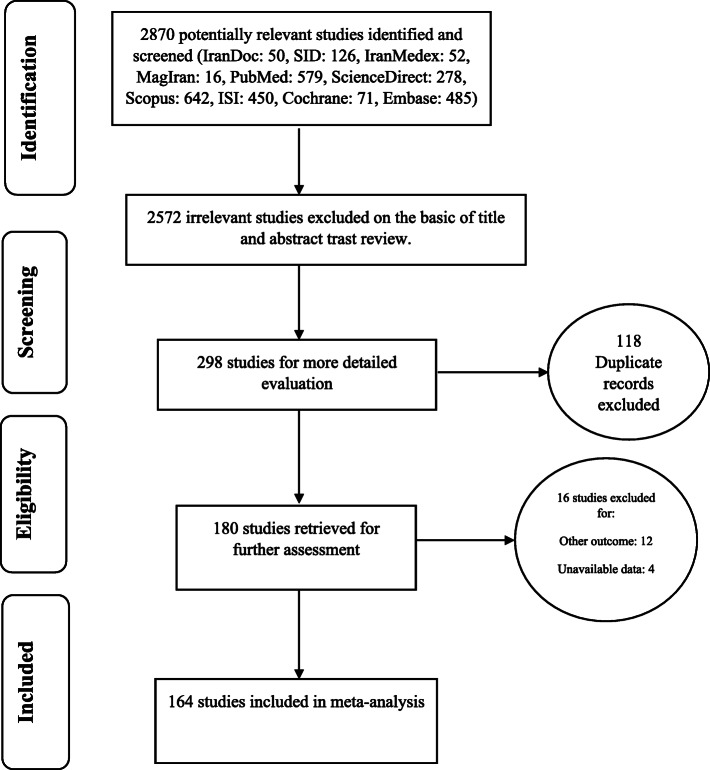
Flow diagram of study selection

In this study, all studies conducted on the prevalence of primary and permanent dental caries in children in the world were systematically investigated without time limitation based on the PRISMA guidelines. In the initial search, 2870 articles were identified, form which 164 studies published between 1995 and December 2019 were eventually entered the final analysis (Fig. 1).

The total sample size for the prevalence of primary dental caries was 80,405 and for the prevalence of permanent dental caries was 1,454,871, given with the mean age of the subjects in each study. The specifications of the selected articles are presented in Tables 1 and 2.

**Table 1:** Characteristic of included studies prevalence of tooth decay

Author, year, Reference	Age (years)	Country	Sample size	Prevalence %	Quality
Kalantari, 2014, [11]	6–7	Iran	400	63.5	High
Abedini, 2013, [12]	2–6	Iran	310	48.7	High
Hematyar, 2009, [13]	3–7	Iran	200	63.5	High
Nabipour, 2013, [14]	3–6	Iran	838	71.8	High
Pahlavani, 2008, [15]	2–6	Iran	414	61.6	High
Amiri, 2017, [16]	4–6	Iran	359	87.7	High
Ajami, 2005, [17]	6–7	Iran	1938	76.5	High
Javadinezhad, 2008, [18]	40.5 Month	Iran	100	77.0	High
Karimi, 2012, [19]	2–5	Iran	211	82.9	High
Amanlou, 2011, [20]	3–6	Iran	205	49.3	High
Toutouni, 2015, [21]	2–3	Iran	239	61.1	High
Bagherian, 2013, [22]	2–5	Iran	400	51.3	Medium
Mohebbi, 2006, [23]	1–3	Iran	504	32.9	High
Ramos-Gomez, 1995, [24]	<6	USA	220	30.0	High
Rosenblatt, 2002, [25]	1–3	USA	468	28.4	High
Rajab, 2001, [26]	1–5	Jordan	384	47.9	High
Douglass, 1999, [27]	3–4	USA	517	37.9	High
Hallett, 1998, [28]	4–6	Australia	3375	37.6	High
Sayegh, 2002, [29]	4–5	Jordan	1140	67.0	Medium
Hallett, 2003, [30]	4–5	Australia	2474	37.6	High
Peressini, 2000, [31]	3–5	Canada	87	19.5	High
Chadwick, 2005, [32]	2–5	UK	449	24.1	High
Schroth, 2015, [33]	<6	Canada	408	53.7	High
Tsai, 1997, [34]	<6	Taiwan	951	55.9	Medium
Mahejabeen, 2006, [35]	3–5	India	1500	54.1	Medium
Du, 2002, [36]	3–5	China	2014	55.3	High
Ferro, 2005, [37]	3–5	Italy	4198	25.0	High
Schroth, 2004, [38]	1–5	USA	834	71.0	Medium
Wyne, 2008, [39]	3–5	Saludi Arabia	789	74.8	High
Lawrence, 2004, [40]	1–5	Canada	1275	72.7	High
Vazquez-Nava, 2005, [41]	4–5	Mexico	1160	17.9	High
Jigjid, 2005, [42]	1–5	Japan	670	71.9	High
Senesombath, 2010, [43]	36–47 Month	Thailand	400	82.0	Medium
Slabsinskiene, 2003, [44]	3	Lithuania	950	50.6	High
Zhou, 2010, [45]	32 Month	China	155	28.4	High
Rajshekar, 2005, [46]	1–6	India	500	43.4	Medium
Ozer, 2011, [47]	3–6	Turkey	226	49.6	High
Li, 2011, [48]	36–70 Month	China	1523	56.8	High
Kumarihamy, 2011, [49]	1–2	Sri Lanka	410	32.2	High
Prakash, 2012, [50]	8–48 Month	India	1500	27.5	Medium
Singh, 2012, [51]	36–60 Month	India	717	40.0	High
Perera, 2010, [52]	24–71 Month	Sri Lanka	410	32.2	Medium
Phipps, 2011, [53]	12–71 Month	India	8461	62.3	High
Parisotto, 2012, [54]	36–59 Month	Brazil	351	39.9	High
Zhang, 2012, [55]	5	China	723	84.9	High
Tanaka, 2007, [56]	3	Japan	2055	20.7	Medium
Colombo, 2019, [57]	48–71 Month	Italy	2522	38.0	Medium
Agouropoulos-1, 2019, [58]	<7	USA	175	92.6	High
Agouropoulos-2, 2019, [58]	<7	USA	175	53.7	High
Agouropoulos-3, 2019, [58]	<7	USA	175	36.0	High
Musinguzi, 2019, [59]	3–5	Kenya	432	48.1	High
Montes, 2019, [60]	5–7	Brazil	415	42.9	Medium
Boustedt, 2019, [61]	5	Sweden	336	13.1	High
Tonpe-1, 2019, [62]	3–5	India	358	2.8	High
Tonpe-2, 2019, [62]	3–5	India	358	4.2	High
Wang, 2019, [63]	6	China	4936	87.7	High
Nomura, 2019, [64]	5–6	Myanmar	187	81.3	Medium
Wu, 2019, [65]	5–6	China	1350	51.4	High
Abbass, 2019, [66]	5–6	Egypt	369	4.3	Medium
Goenka, 2018, [67]	5–7	India	312	65.1	High
Chugh, 2018, [68]	24–61 Month	India	425	47.3	High
Vandana, 2018, [69]	2–6	India	550	38.2	High
Igic, 2018, [70]	3–6	Serbia	250	38.4	High
Kato, 2017, [71]	3	Japan	6315	36.0	High
Li, 2017, [72]	3–5	China	1727	78.2	High
Mangla, 2017, [73]	1–3	India	510	21.0	High
Owen, 2017, [74]	3–5	Australia	623	14.1	High
Pal, 2017, [75]	5–6	India	408	46.6	High
Wagne, 2017, [76]	6.7	Germany	512	1.8	High
Shah, 2017, [77]	5–7	India	829	33.2	Medium
Yuan, 2017, [78]	3	China	959	28.1	High
Jiang, 2017, [79]	2–5	China	1509	71.4	High
Massignan, 2016, [80]	3.7	Brazil	565	39.1	High
Koya, 2016, [81]	24–71 Month	India	1897	42.0	High
Mothupi, 2016, [82]	4.8	Africa	495	48.9	High
Alkhtib, 2016, [83]	4–5	Qatari	250	89.2	High
Henry, 2016, [84]	<3	India	1486	40.6	High
Šačić, 2016, [85]	3–5	Bosnia and Herzegovina	165	17.0	Medium
Al-Meedani, 2016, [86]	3–5	Saludi Arabia	388	69.1	High
Elidrissi, 2016, [87]	3–5	Sudan	553	52.4	Medium
Gopal, 2016, [88]	3–6	India	477	27.3	High

**Table 2:** Characteristic of included studies Prevalence of permanent dental caries

Author, year, Reference	Age (years)	Country	Sample size	Prevalence %	Quality
Aghighi, 2010, [89]	6–15	Iran	4666	66.3	High
Mortazavi, 1997, [90]	6–9	Iran	220	65.5	High
Asdagh, 2015, [91]	6–12	Iran	847	79.7	Medium
Memar, 1999, [92]	12	Iran	439	84.3	High
Javadi nejad, 2006, [93]	12	Iran	340	82.1	High
Sadeghi, 2007, [94]	12	Iran	563	68.6	High
Yousofi, 2015, [95]	7–12	Iran	460	89.8	High
Eskandarizadeh, 2015, [96]	6–12	Iran	15,369	79.5	High
Mossaheb, 2011, [97]	6–11	Iran	203	82.3	High
Qin, 2019, [98]	10–12	China	5057	39.2	High
Alshehhi, 2019, [99]	8.1	United Arab Emirates	62	58.1	High
Cheng, 2019, [100]	10.3	China	1,196,004	41.1	High
Villanueva-Gutiérrez, 2019, [101]	9	Spain	686	35.4	High
Lešić, 2019, [102]	6–15	Croatia	1589	50.0	High
Mohd Nor, 2019, [103]	12	Malaysia	595	74.3	High
Vanvitelli, 2019, [104]	8–10	Italy	530	29.1	Medium
Obregón-Rodríguez-1, 2019, [105]	12	Spain	1045	25.5	High
Obregón-Rodríguez-2, 2019, [105]	15	Spain	783	26.2	High
Mimoza, 2019, [106]	7–10	Italy	398	28.4	Medium
Abbass, 2019, [66]	6–12	Egypt	369	27.9	High
Aldossary, 2018, [107]	6–9	Saudi Arabia	1844	95.0	High
Goenka, 2018, [67]	8–10	India	353	56.7	High
Ballouk, 2019, [108]	8–12	Syria	1500	79.1	High
Alhabdan, 2018, [109]	6–8	Saudi Arabia	578	82.9	High
Konde, 2018, [110]	12	India	1000	13.6	High
Solis-Riggioni, 2018, [111]	8–15	Costa Rica	201	35.8	High
Musa, 2018, [112]	7–11	China	24,521	32.4	High
Dutra, 2018, [113]	8–12	Brazil	1211	32.4	High
Al-Akwa, 2018, [114]	6–12	Yemen	17,599	67.6	Medium
Cruz, 2018, [115]	11–12	Brazil	184	34.2	High
Andegiorgish, 2017, [116]	12	Eritrea	225	77.8	High
Alwayli, 2017, [117]	6–9	Saudi Arabia	17,891	64.6	High
Dobbiani-1, 2012, [118]	10	Italy	400	44.0	Medium
Dobbiani-2, 2013, [118]	10	Italy	400	18.5	Medium
Maran, 2017, [119]	6–12	India	1204	73.2	High
Shah, 2017, [77]	12–15	India	829	31.4	High
Kim, 2017, [120]	6–11	Korea	514	49.4	High
Sköld, 2016, [121]	13	Sweden	758	2.6	High
Plaka, 2017, [122]	12–15	India	193	36.3	High
Hiremath, 2016, [123]	6–11	India	13,200	78.9	High
Kottayi, 2016, [124]	12–15	India	2000	3.9	Medium
Ponnudurai, 2016, [125]	6–14	India	2796	68.8	High
Djossou, 2013, [126]	6–15	Benin	497	49.7	High
Weusmann, 2015, [127]	8	Germany	25,020	60.9	High
Goel-1, 2015, [128]	12	India	992	34.3	Medium
Goel-2, 2015, [128]	15	India	992	46.5	High
Farooqi, 2014, [129]	6–9	Saudi Arabia	711	73.0	High
Arora, 2014, [130]	12	India	100	57.0	High
Sukhabogi-1, 2014, [131]	12	India	924	39.9	Medium
Sukhabogi-2, 2014, [131]	15-	India	951	46.7	Medium
Al-Darwish, 2014, [132]	12–14	Qatar	2113	85.0	High
Aidara, 2014, [133]	12–15	Senegal	677	96.0	High
Ingle, 2014, [134]	12–15	India	1400	53.0	Medium
Sofola, 2000, [135]	6–12	Nigeria	513	16.6	High
Das, 2013, [136]	6–14	West Bengal	1764	28.1	High
Mahfouz-1, 2013, [137]	12	Palestine	677	40.6	High
Mahfouz-2, 2013, [137]	13	Palestine	677	41.8	High
Mahfouz-3, 2013, [137]	14	Palestine	677	60.4	High
Riziwaguli, 2013, [138]	7–9	China	1600	26.5	Medium
Joshi, 2013, [139]	6–12	India	1600	69.1	Medium
Pieper, 2009, [140]	12	Germany	30,943	72.7	High
Yengopal, 2012, [141]	10.5	Africa	882	27.6	High
Murthy, 2014, [142]	12–15	India	1452	57.9	High
Koposova, 2013, [143]	12	Russia	590	68.0	High
Dixit, 2013, [144]	12–13	Nepal	361	41.0	Medium
Suprabha, 2013, [145]	11–13	India	857	59.4	High
Panagidis, 2012, [146]	12	Germany	951	32.6	High
Shailee-1, 2012, [147]	12	India	1011	32.6	Medium
Shailee-2, 2012, [147]	15	India	1011	42.2	High
Lagana, 2012, [148]	7–15	Albanian	2617	88.9	Medium
Subedi, 2011, [148]	12–13	Nepal	325	53.2	Medium
Shekar, 2011, [149]	12–15	India	474	56.3	High
Oulis-1, 2010, [150]	12	Greece	1224	80.0	High
Oulis-2, 2010, [150]	15	Greece	1257	83.0	High
Jamelli, 2010, [151]	12	Brazil	689	71.8	High
Kanagaratnam, 2009, [152]	9	New Zealand	612	54.9	High
Bissar, 2008, [153]	11–14	Germany	570	42.3	High
Ferro, 2007, [154]	12	Italy	260	56.9	High
Moreira, 2006, [155]	12–15	Brazil	1665	50.9	High
Schulte, 2004, [156]	12	Germany	43,950	39.3	High
Paredes, 2005, [157]	6–10	Spain	600	47.2	High
Mestriner, 2005, [158]	12	Brazil	256	53.9	High
Traebert, 2002, [159]	12	Brazil	803	62.1	High

### Heterogeneity and publication bias

**Fig. 2:**
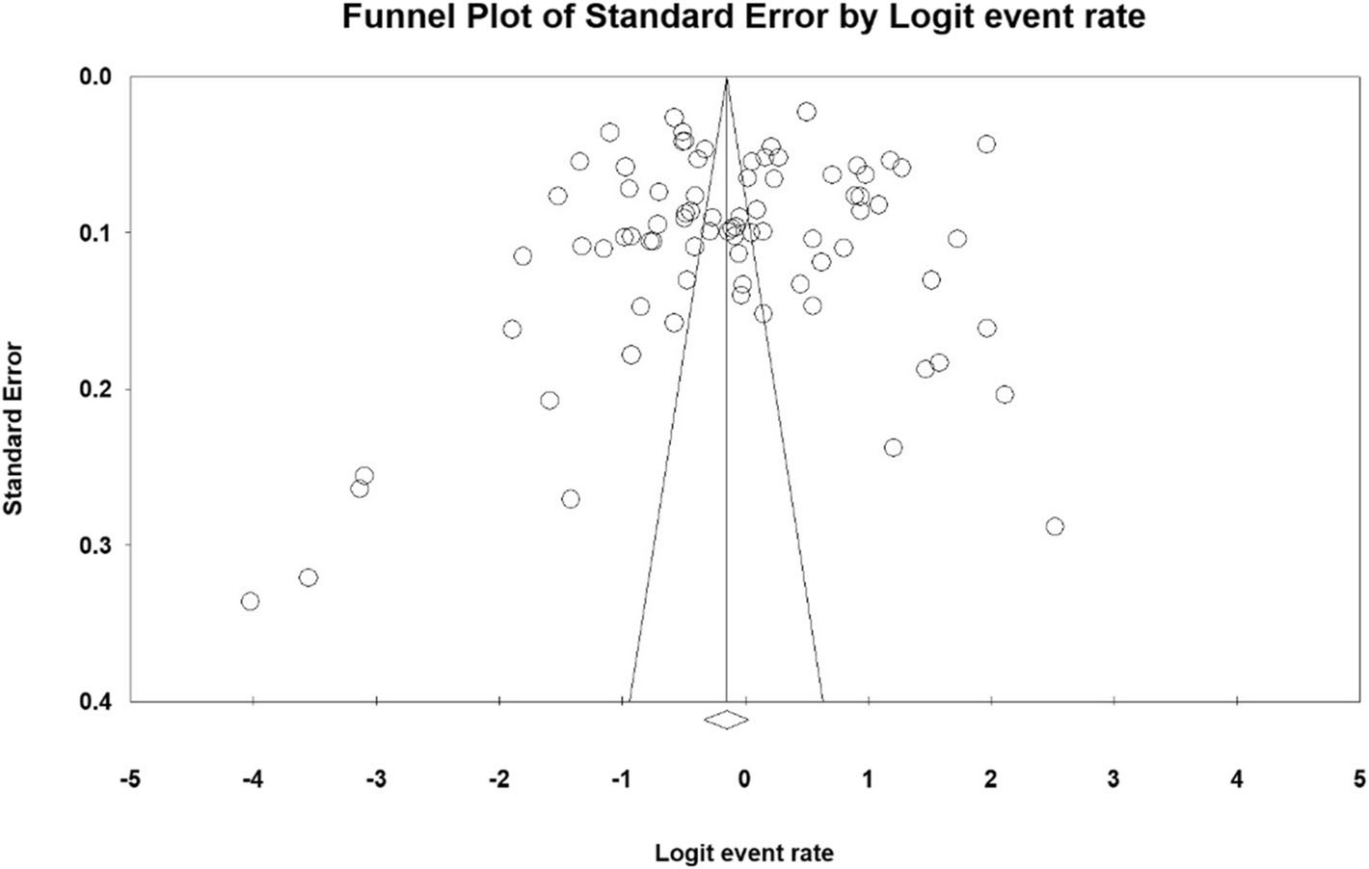
Funnel plot of the results of the prevalence of dental caries in primary teeth in children

**Fig. 3:**
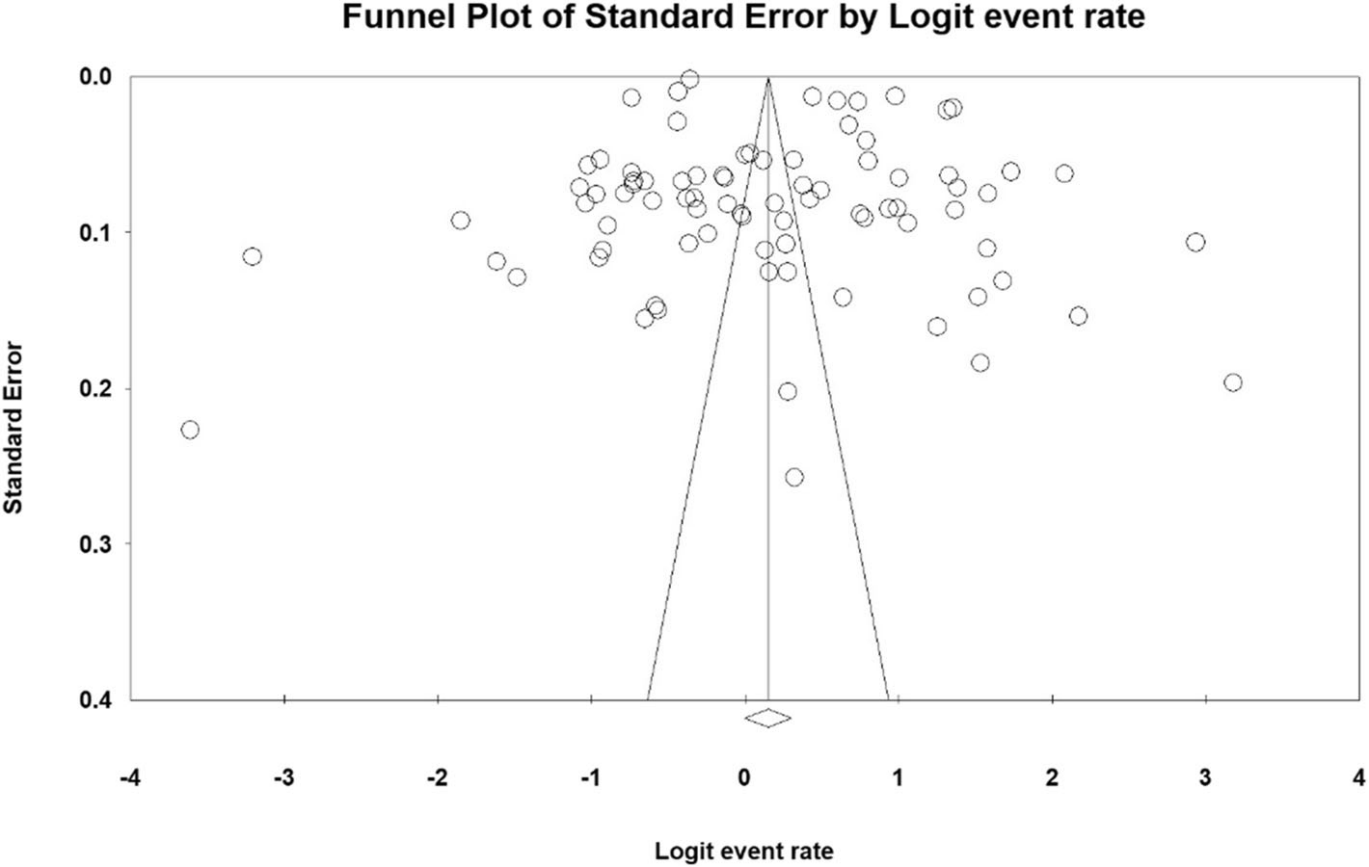
Funnel plot of the results of the prevalence of dental caries in permanent teeth in children

Based on the results of the heterogeneity evaluation test (I^2^), the prevalence of dental caries in primary and permanent teeth was reported to be I^2^: 99.2 and I2: 99.8, respectively. Due to the heterogeneity of the selected studies, a random effects model was used to combine the studies and jointly estimate the prevalence of dental caries in primary and permanent teeth. The probability of publication bias was evaluated by the Funnel plot and the Begg and Mazumdar tests at a significant level of 0.1 (Figs. 2 and 3), indicating that the publication bias was not statistically significant in the investigation of the prevalence of primary dental caries (*P* = 0.590) and permanent dental caries (*P* = 0.145).

### Meta-analysis (primary dental caries)

**Fig. 4:**
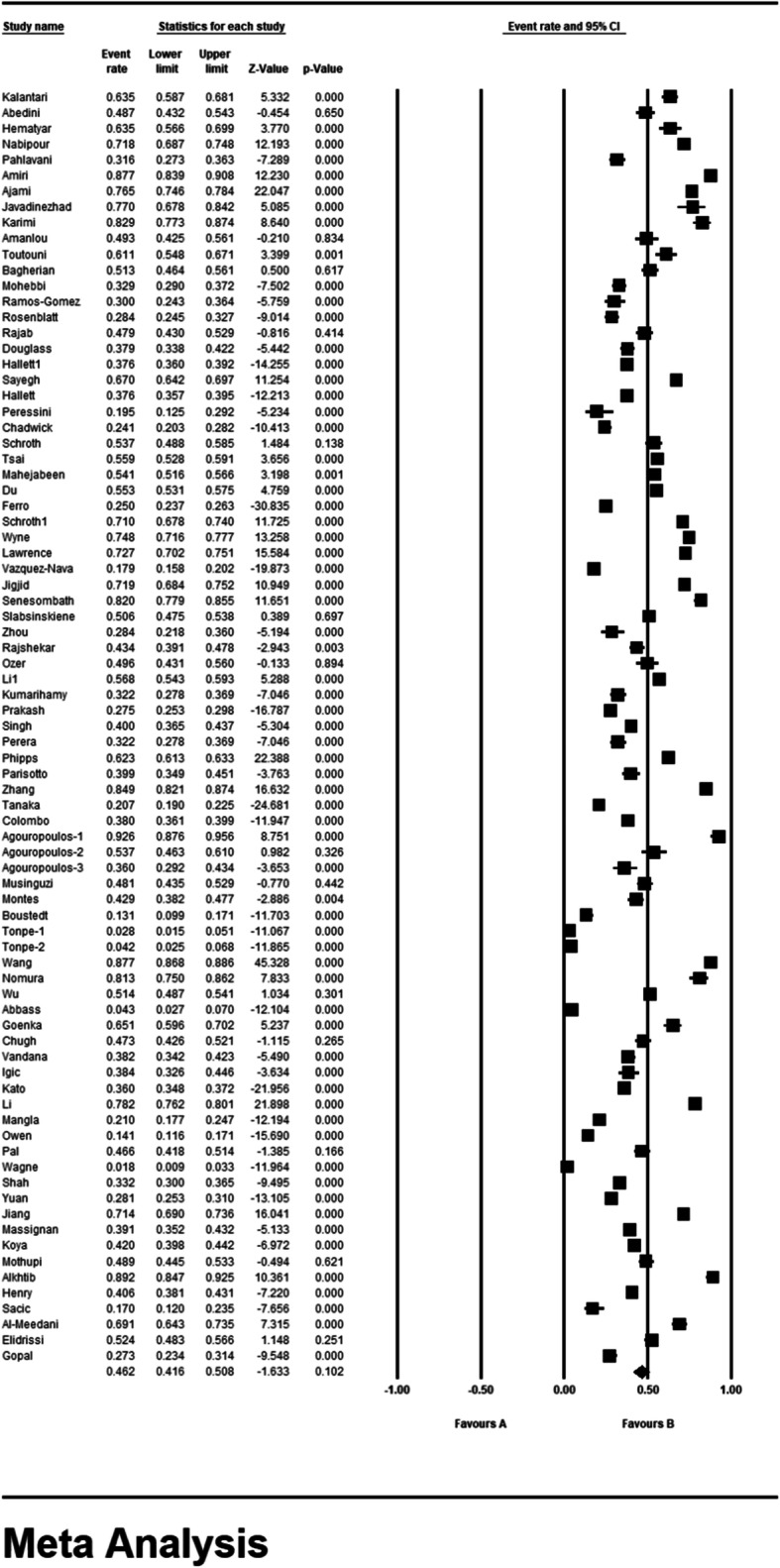
Forest plot of the results of Prevalence of dental caries in primary teeth and 95% confidence intervals worldwide

According to the results of the study in forest plot, the overall prevalence of dental caries in primary teeth in children in the world was 46.2% (95% CI: 41.6–50.8%) (Fig. 4). The middle point of each line shows the prevalence of primary dental caries in the world for each study, and the rhombic figure shows the prevalence of primary dental caries in the world for all studies.

### Meta-analysis (permanent dental caries)

**Fig. 5:**
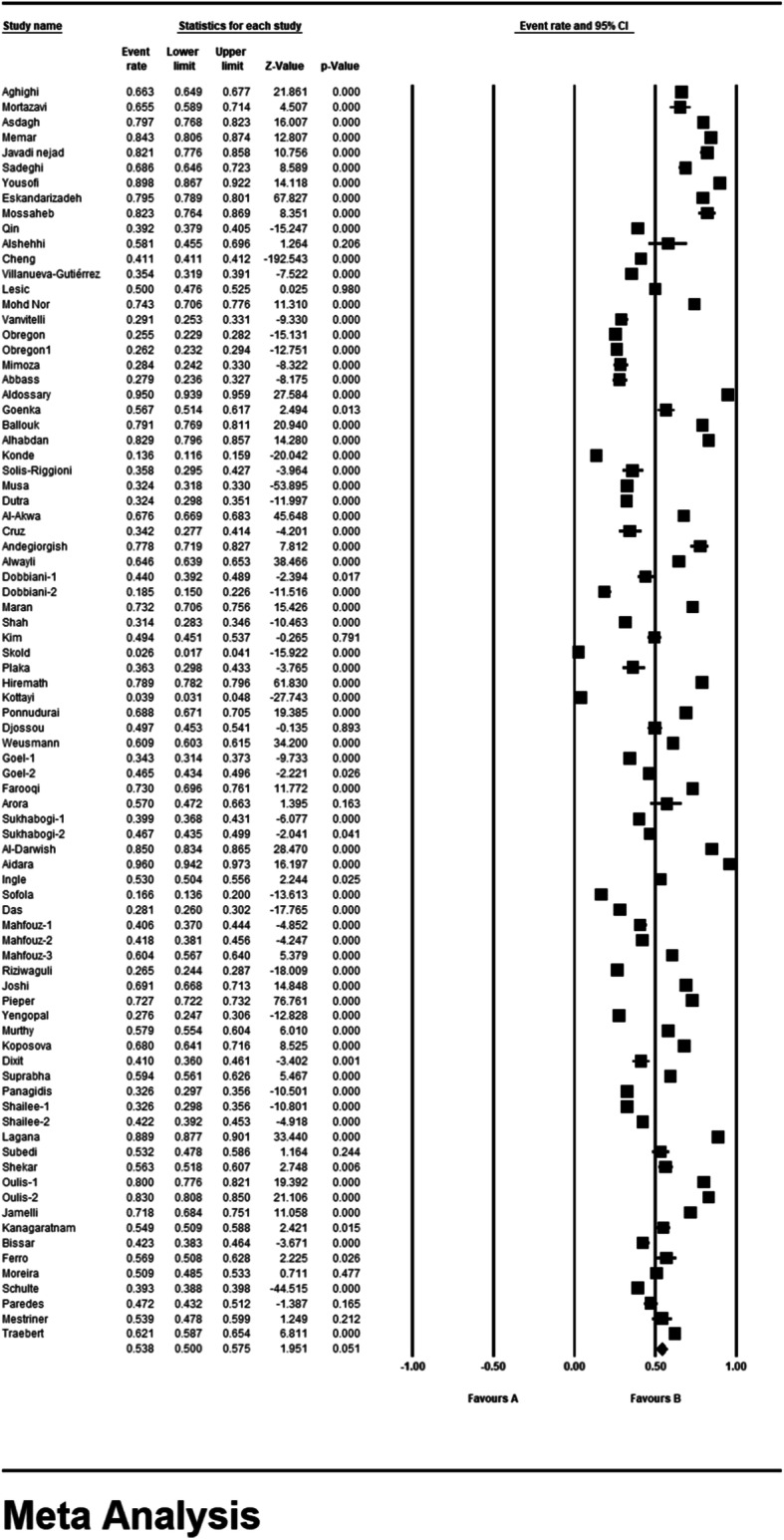
Forest plot of the results of Prevalence of dental caries in permanent teeth and 95% confidence intervals worldwide

According to the results of the study in forest plot, the overall prevalence of dental caries in permanent teeth in children in the world was 53.8% (95% CI: 50–57.5%) (Fig. 5). The middle point of each line shows the prevalence of permanent dental caries in the world for each study, and the rhombic figure shows the prevalence of permanent dental caries in the world for all studies.

### Meta-regression

**Fig. 6:**
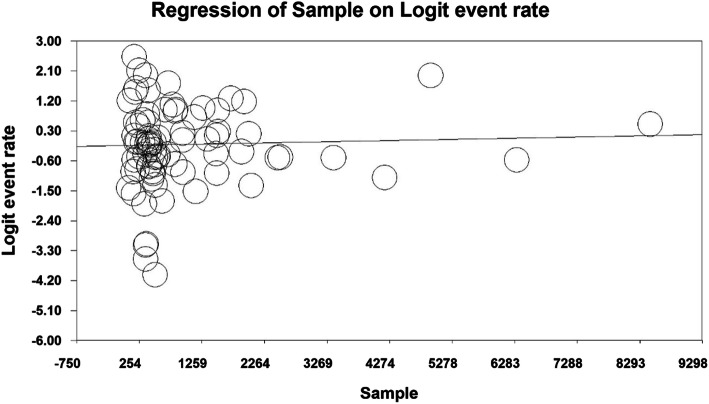
Meta-regression analysis of the relationship between the sample size and the prevalence of dental caries in primary teeth in children in the world

**Fig. 7:**
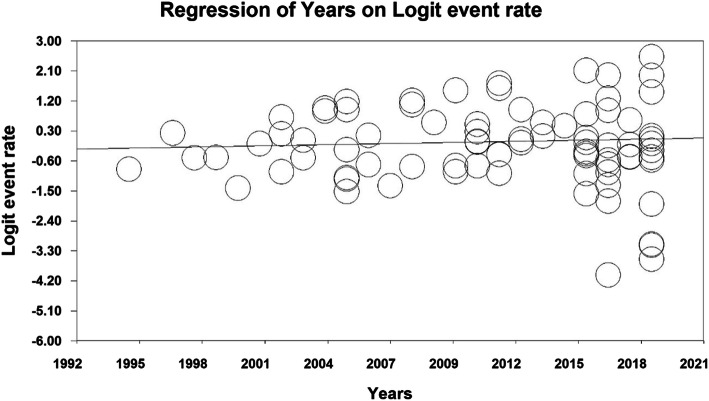
Meta-regression analysis of the relationship between the year of study and the prevalence of dental caries in primary teeth in children in the world

The prevalence of primary dental caries was evaluated by meta-regression analysis based on the year of study and the sample size, which reported that with increasing the year of study and the sample size, the prevalence of dental caries in primary teeth increased in both cases and the difference was statistically significant (P < 0.01) (Figs. 6 and 7).

**Fig. 8:**
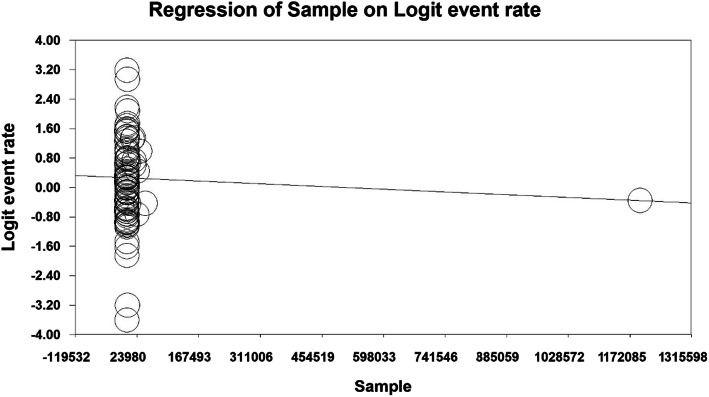
Meta-regression analysis of the relationship between the sample size and the prevalence of dental caries in permanent teeth in children in the world

**Fig. 9:**
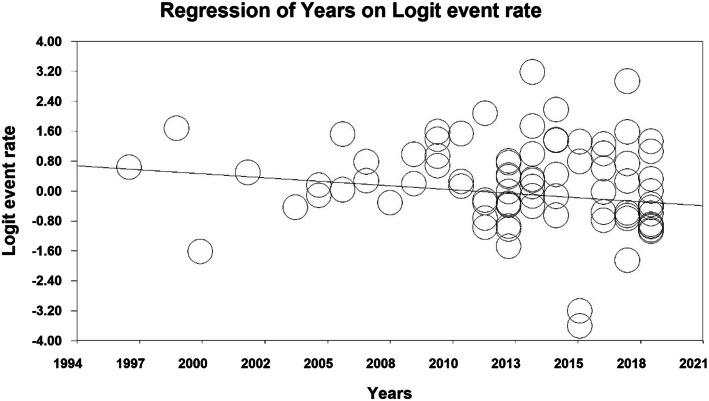
Meta-regression analysis of the relationship between the year of study and the prevalence of dental caries in permanent teeth in children in the world

The prevalence of permanent dental caries was evaluated based on the year of study and the sample size, which reported that with increasing the year of study and the sample size, the prevalence of dental caries in permanent teeth decreased in both cases and the difference was statistically significant (P < 0.01) (Figs. 8 and 9).

### Sub-group analysis

**Fig. 10:**
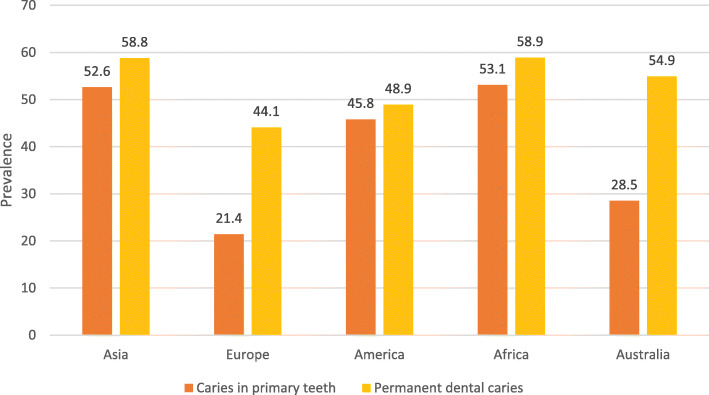
Providing the results of the prevalence of dental caries in primary and permanent teeth in children in different continents

Also, Table 3 and Fig. 10 report the results of the prevalence of dental caries in primary and permanent teeth in children in different continents. These changes were reported in the continents of Asia, Europe, Africa, USA, and Australia, according to which 100 Asian studies (50 studies on the prevalence of dental caries in primary teeth and 50 studies on the prevalence of dental caries in permanent teeth), 32 European studies (10 studies on the prevalence of dental caries in primary teeth and 22 studies on the prevalence of dental caries in permanent teeth), 21 American studies (14 studies on the prevalence of dental caries in primary teeth and 7 studies on the prevalence of dental caries in permanent teeth), 10 African studies (5 studies on the prevalence of dental caries in primary teeth and 5 studies on the prevalence of dental caries in permanent teeth), and 4 Australian studies (3 studies on the prevalence of dental caries in primary teeth and 1 studies on the prevalence of dental caries in permanent teeth) were included in the meta-analysis. There was a significant difference in the prevalence of dental caries in primary and permanent teeth in different continents (Table 3 and Fig. 10).

**Table 3:** Investigating the prevalence of dental caries in primary and permanent teeth in children in different continents

Tooth type	continents	Number of articles	Sample Size	I^**2**^	Egger Test	Prevalence %
Caries in primary teeth	Asia	50	54,680	99.3	0.756	52.6 (95% CI: 46.7–58.5)
	Europe	10	9977	98.4	0.152	21.4 (95% CI: 15.3–29.1)
	America	14	6825	98.7	0.742	45.8 (95% CI: 34.2–58)
	Africa	5	3004	95.5	0.220	53.1 (95% CI: 44.3–61.7)
	Australia	3	6472	98.3	0.296	28.5 (95% CI: 20.3–38.5)
Permanent dental caries	Asia	50	1,334,133	99.8	0.284	58.8 (95% CI: 53.4–64)
	Europe	22	115,141	99.8	0.175	44.1 (95% CI: 36.1–52.5)
	America	7	5009	98.2	0.763	48.9 (95% CI: 37.6–60.3)
	Africa	5	2794	99.3	0.220	58.9 (95% CI: 29.4–83.1)
	Australia	1	612	–	–	54.9 (95% CI: 50.9–58.8)

### Cumulative meta-analysis

**Fig. 11:**
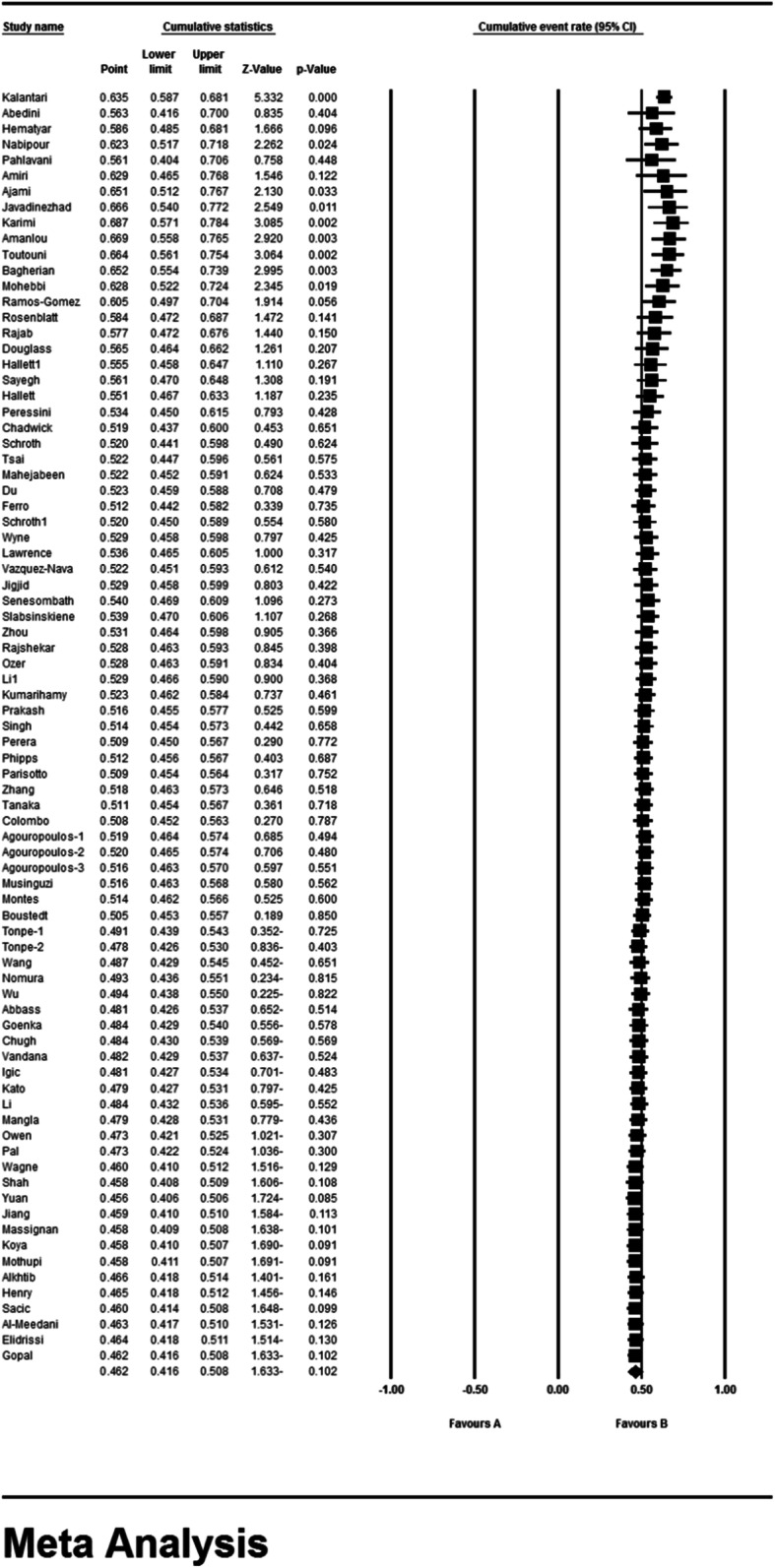
Result of cumulative meta-analysis based on primary dental caries

**Fig. 12:**
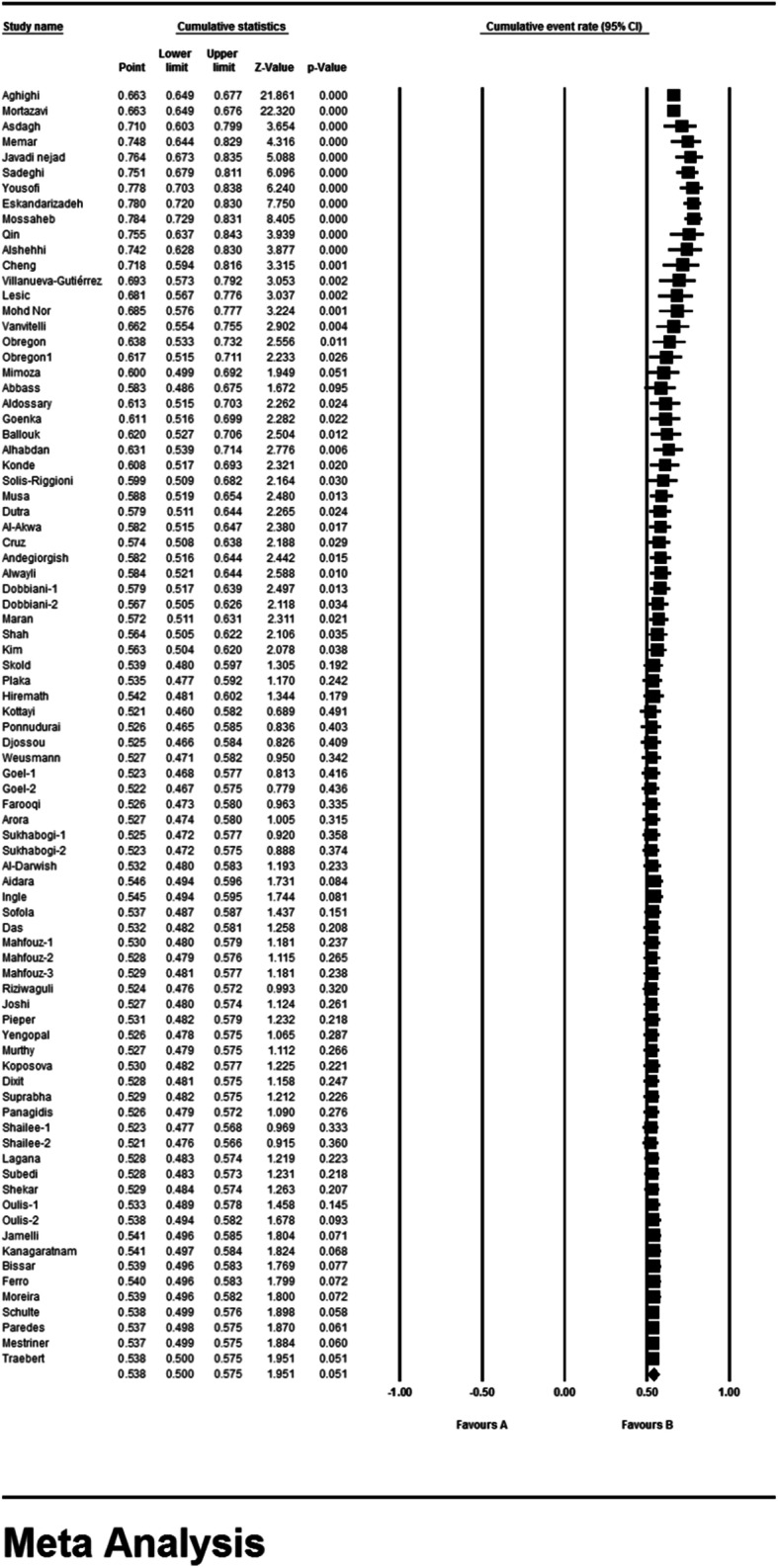
Result of cumulative meta-analysis based on permanent dental caries

A cumulative meta-analysis of the included studies was performed based primary and permanent dental caries. Cumulative risk of each study’s addition to the meta-analysis are reported in Figs. 11 and 12.

## Discussion

In this study, the prevalence of dental caries in studies conducted throughout the world was investigated, and it was reported that the overall prevalence of dental caries in primary teeth in children was 46.2%. Early childhood caries (ECC) in developing countries was reported to be more than in developed countries [1]. Also, in the present study, the overall prevalence of dental caries in permanent teeth in children was 53.8%.

Differences in the prevalence of dental caries in developed and underdeveloped countries may be due to differences in the age groups studied, but may also be due to ethnic, cultural, geographic, racial, and developmental differences as well as access to dental services, behavioral habits, health care behaviors, nutritional habits and behaviors, and lifestyle [144]. The effects of parents’ lack of awareness of their children’s tooth decay status as well as neglect and attention discrimination can also be well documented in the study of Nag et al. [160], suggesting that in the age group of 6 to 18 years, caries rates were higher in girls than in boys, as girls are more neglected by parents than boys. Although there has been no difference in the prevalence rate of both sexes in the current systematic study, terms of access to health services and lack of parental awareness of and attention to children’s dental caries are known as the most important factors in its development [2], so discrimination and inequality in the upbringing of children in the family can also multiply the impact of such a situation.

In the present meta-analysis study, the prevalence of dental caries in primary and permanent teeth in different continents is presented in Table 3 and Fig. 10, which was higher in the African continent and secondarily in the Asian continent.

According to the World Health Organization’s Healthy People Plan, 90% of children between the ages of 5 and 6 should be free of tooth decay by 2010. However, according to the findings of this study which were based on the reviews performed in the studies searched, the prevalence of dental caries in children in most countries was found to be very high. Such a situation gives rise to worrying conditions in terms of tooth decay in adulthood and will also impose enormous tooth repair costs on the country’s health sector. Such a situation in the country, in addition to what has been said, as well as conditions such as inequality in access to health care services, inequality in developmental and economic situation in different countries and different parts of countries may indicate that the lack of awareness of the health and preservation of primary teeth in all families with different socioeconomic status has been considered a serious problem [3] and a barrier to the provision of preventive and health services.

Families and parents should know that child dental care must start from the mother’s pregnancy; children born to mothers with multiple dental caries are more likely to develop caries in the later stages of their lives. Cariogenic bacteria are usually transferred through the use of a spoon or a bottle of milk from the mother’s moth to the child’s mouth for the first time, so breast feeding should be avoided as much as possible during the baby’s sleep. Regular dental appointments should be provided from the beginning of the baby’s primary teeth eruption, especially with the eruption of the first permanent tooth, first molar tooth or the 6the tooth. The tooth develops immediately after the last primary tooth at the age of six and is most likely to be decayed. The American Academy of Pediatric Dentistry, the American Dental Association, and the American Academy of General Dentistry all recommend making sure to see a dentist 6 months after the eruption of teeth in children, before the age of one year’s [161–166]. One of the most important and available measures to prevent caries, especially primary teeth in children, is performing dental procedures such as fissure sealant and fluoride therapy. In the fissure sealant method, deep grooves in the surface of the tooth are covered with a thin layer of tooth-colored material, thereby preventing the spread of cariogenic bacteria in tooth grooves. Other methods are caries prevention [167]and[168].

Given the high prevalence of primary and permanent dental caries in children worldwide, it is recommended that providing educational programs and interventions in primary and permanent dental health care especially for mothers, nurses, and child educators be of special interest to health services policy-makers and providers. Planning to provide educational programs and inexpensive dental and oral health services as well as ease of access to such services for children by the health system of countries is noted as well.

### Strength and limitation

The most important strength of this study is that it has been studied for the first time in the world, includes all data sources and high-quality studies, and also analysis based on different continents for the use of the World Health Organization. The most important limitation of the present study is inaccessibility to the full text of the articles, incomplete search, and poor quality of some studies, as well as restricted search based on Persian and English languages.

## Conclusion

The results of this study showed that the prevalence of dental caries in primary and permanent teeth in children in the world was found to be high. Therefore, appropriate strategies should be implemented to improve the aforementioned situation and to troubleshoot and monitor at all levels by providing feedback to hospitals. Also, the prevalence of dental caries in primary and permanent teeth in children of Africa is higher than other continents and requires special attention of the World Health Organization to this continent in improving the oral health of children. These strategies can include providing educational programs to parents, periodic dental care for children, and fluoride therapy in childhood for the African continent.

## Data Availability

Datasets are available through the corresponding author upon reasonable request.

## Abbreviations

ECC: Early Childhood Caries
WHO: World Health Organization
DMF: Decay-Missing-Filled
SID: Scientific Information Database
MESH: Medical Subject Headings
ISI: Web of Science
PRISMA: Preferred Reporting Items for Systematic Reviews and Meta-Analysis
STROBE: Strengthening the Reporting of Observational Studies in Epidemiology for cross- sectional Study

## References

[1] Nematollahi H, Mehrabkhani M, Sheykhani M (2007). Assessing the relationship between diet and prevalence of early childhood caries in Birjand preschool children. J Dent.

[2] Wagle M, D'Antonio F, Reierth E, Basnet P, Trovik TA, Orsini G, Manzoli L, Acharya G (2018). Dental caries and preterm birth: a systematic review and metaanalysis. BMJ Open.

[3] Broumand S, Sharififar S, Alikhani S (2006). The study of caries free indicator of milk teeth in children age 3–6 at dare care center affiliated to health centers of Army.

[4] Moynihan P, Petersen PE (2004). Diet, nutrition and the prevention of dental diseases. Public Health Nutr.

[5] Hallett KB, O'Rourke PK (2006). Pattern and severity of early childhood caries. Community Dent Oral Epidemiol.

[6] McDonald RE, Avery DR (2004). Dentistry for the child and adolescent, Mosby Incorporated.

[7] Bayat Movahed S, Samadzadeh H, Ziyarati L, Memory N, Khosravi R, Eshkevari PS (2011). Oral health of Iranian children in 2004: a national pathfinder survey of dental caries and treatment needs.

[8] World Health Organization (1997). Oral health surveys-basic methods.

[9] Vanobbergen J, Lesaffre E, Garcia-Zattera M, Jara A, Martens L, Declerck D (2007). Caries patterns in primary dentition in 3-, 5-and 7-year-old children: spatial correlation and preventive consequences. Caries Res.

[10] Vandenbroucke JP, VON Elm E, Altman DG, Gotzsche PC, Mulrow CD, Pocock SJ (2007). Strengthening the reporting of observational studies in epidemiology (STROBE): explantion and elaboration. Epidemiology.

[11] Kalantari B, Rahmannia J, Hatami H, Karkhaneh S, Farsar A, Sharifpoor A, Zahedi B (2014). The prevalence of dental caries in primary molars and its related factors in 6 and 7 years old children in Shemiranat health center. J Health Field.

[12] Abedini H (2013). Prevalence and causes of decay in primary teeth of children aged 2–6 years in Kashan.

[13] Hematyar M, Masnavi A (2009). Prevalence and risk factors of dental decays in 3-7 years old children referred to pediatric clinics of Islamic Azad University. J Qazvin Univ Med Sci.

[14] Nabipour AR, Azvar K, Zolala F, Ahmadinia H, Soltani Z (2013). The prevalence of early dental caries and its contributing factors among 3-6-year-old children in Varamin/Iran. Health Dev J.

[15] Pahlavani Z, Egjbalian F, Moksefesfahani F, Chitgar Z (2008). Determination of frequency and pattern of dental caries and its effective factors in 2 to 6 year old children in Hamedan. Feyz Sci J.

[16] Amiri S, Veissi M, Saleki M, Rahmani M, Haghighizadeh M (2017). The relationship between dental caries with dietary habits and body mass index in 4–6 years old kindergartens in Ahvaz.

[17] Ajami B, Ebrahimi M (2005). Evaluation of oral health status amongst 6-7-year-old children in Mashhad in 2001. J Mashhad Dental Sch.

[18] Javadinezhad SH, Karami M, Fayz E (2008). Nutritional habits and the prevalence of dental caries in preterm or low birth weight children. J Shiraz Dent.

[19] Karimi Shahanjarini A, Makvandi Z, Faradmal J, Bashirian S, Hazavehei M (2012). Assessing the tooth decay status of 2-5 years children and the role of their mothers’ caring behaviors. Sci J Hamadan Nurs Midwifery Faculty.

[20] Amanlou M, Jafari S, Afzalianmand N, Omrany ZB, Farsam H, Nabati F, Bagherzadeh K (2011). Association of Saliva Fluoride level and socioeconomic factors with dental caries in 3-6 years old children in Tehran-Iran. Iran J Pharm Res.

[21] Toutouni H, Nokhostin M-R, Amaechi BT, Zafarmand AH (2015). The prevalence of early childhood caries among 24 to 36 months old children of Iran: using the novel ICDAS-II method. J Dent.

[22] Bagherian A, Sadeghi M (2013). Association between dental caries and age-specific body mass index in preschool children of an Iranian population. Indian J Dent Res.

[23] Mohebbi SZ, Virtanen JI, Vahid-Golpayegani M, Vehkalahti MM (2006). Early childhood caries and dental plaque among 1-3-year-olds in Tehran, Iran. J Indian Soc Pedod Prev Dent.

[24] Ramos-Gomez FJ, Tomar SL, Ellison J, Artiga N, Sintes J, Vicuna G (1999). Assessment of early childhood caries and dietary habits in a population of migrant Hispanic children in Stockton, California. ASDC J Dent Child.

[25] Rosenblatt A, Zarzar P (2002). The prevalence of early childhood caries in 12-to 36-month-old children in Recife, Brazil. J Dent Child.

[26] Rajab LD, Hamdan M (2002). Early childhood caries and risk factors in Jordan. Community Dent Health.

[27] Douglass J, Montero M, Thibodeau E, Mathieu G (2002). Dental caries experience in a Connecticut head start program in 1991 and 1999. Pediatr Dent.

[28] Hallett KB, O'Rourke PK (2002). Early childhood caries and infant feeding practice. Community Dent Health.

[29] Sayegh A, Dini E, Holt R, Bedi R (2002). Caries in preschool children in Amman, Jordan and the relationship to socio‐demographic factors. Int Dent J.

[30] Hallett K, O'Rourke P (2003). Social and behavioural determinants of early childhood caries. Aust Dent J.

[31] Peressini S, Leake JL, Mayhall JT, Maar M, Trudeau R (2004). Prevalence of early childhood caries among first nations children, district of Manitoulin, Ontario. Int J Paediatr Dent.

[32] Chadwick BL, Treasure ET, Playle RA (2005). A randomised controlled trial to determine the effectiveness of glass ionomer sealants in pre-school children. Caries Res.

[33] Schroth RJ, Pang JL, Levi JA, Martens PJ, Brownell MD (2014). Trends in pediatric dental surgery for severe early childhood caries in Manitoba, Canada. J Can Dent Assoc.

[34] Tsai AI, Chen CY, Li LA, Hsiang CL, Hsu KH (2006). Risk indicators for early childhood caries in Taiwan. Community Dent Oral Epidemiol.

[35] Mahejabeen R, Sudha P, Kulkarni S, Anegundi R (2006). Dental caries prevalence among preschool children of Hubli: Dharwad city. J Indian Soc Pedod Prev Dent.

[36] Du M, Luo Y, Zeng X, Alkhatib N, Bedi R (2007). Caries in preschool children and its risk factors in 2 provinces in China. Quintessence Int.

[37] Ferro R, Besostri A, Olivieri A, Stellini E, Mazzoleni S (2007). Preschoolers’ dental caries experience in and its trend over 20 years in a north-east Italian health district. Eur J Paediatr Dent.

[38] Schroth RJ, Cheba V (2007). Determining the prevalence and risk factors for early childhood caries in a community dental health clinic. Pediatr Dent.

[39] Wyne A (2008). Caries prevalence, severity, and pattern in preschool children. J Contemp Dent Pract.

[40] Lawrence HP, Binguis D, Douglas J, McKeown L, Switzer B, Figueiredo R, Laporte A (2008). A 2‐year community‐randomized controlled trial of fluoride varnish to prevent early childhood caries in Aboriginal children. Community Dent Oral Epidemiol.

[41] Vázquez-Nava F, Vázquez R, Saldivar G, Beltrán G, Almeida A, Vázquez R (2008). Allergic rhinitis, feeding and oral habits, toothbrushing and socioeconomic status. Caries Res.

[42] Jigjid B, Ueno M, Shinada K, Kawaguchi Y (2009). Early childhood caries and related risk factors in Mongolian children. Community Dent Health.

[43] Senesombath S, Nakornchai S, Banditsing P, Lexomboon D (2010). Early childhood caries and related factors in Vientiane, Lao PDR.

[44] Slabšinskienė E, Milčiuvienė S, Narbutaitė J, Vasiliauskienė I, Andruškevičienė V, Bendoraitienė E-A, Saldūnaitė K (2010). Severe early childhood caries and behavioral risk factors among 3-year-old children in Lithuania. Medicina.

[45] Zhou Y, Lin H, Lo E, Wong M (2011). Risk indicators for early childhood caries in 2‐year‐old children in southern China. Aust Dent J.

[46] Rajshekar SA, Laxminarayan N (2011). Comparison of primary dentition caries experience in pre-term low birth-weight and full-term normal birth-weight children aged one to six years. J Indian Soc Pedod Prev Dent.

[47] Ozer S, Sen Tunc E, Bayrak S, Egilmez T (2011). Evaluation of certain risk factors for early childhood caries in Samsun, Turkey. Eur J Paediatr Dent.

[48] Li Y, Zhang Y, Yang R, Zhang Q, Zou J, Kang D (2011). Associations of social and behavioural factors with early childhood caries in Xiamen city in China. Int J Paediatr Dent.

[49] Kumarihamy SL, Subasinghe LD, Jayasekara P, Kularatna SM, Palipana PD (2011). The prevalence of early childhood caries in 1-2 yrs olds in a semi-urban area of Sri Lanka. BMC Res Notes.

[50] Prakash P, Subramaniam P, Durgesh B, Konde S (2012). Prevalence of early childhood caries and associated risk factors in preschool children of urban Bangalore, India: a cross-sectional study. Eur J Dent.

[51] Singh S, Vijayakumar N, Priyadarshini H, Shobha M (2012). Prevalence of early childhood caries among 3-5 year old pre-schoolers in schools of Marathahalli, Bangalore. Dental Res J.

[52] Perera PJ, Abeyweera NT, Fernando MP, Warnakulasuriya TD, Ranathunga N (2012). Prevalence of dental caries among a cohort of preschool children living in Gampaha district, Sri Lanka: a descriptive cross sectional study. BMC Oral Health.

[53] Phipps KR, Ricks TL, Manz MC, Blahut P (2012). Prevalence and severity of dental caries among American Indian and Alaska native preschool children. J Public Health Dent.

[54] Parisotto TM, Steiner‐Oliveira C, De Souza‐e‐Silva CM, Peres RC, Rodrigues LK, Nobre‐Dos‐Santos M (2012). Assessment of cavitated and active non‐cavitated caries lesions in 3‐to 4‐year‐old preschool children: a field study. Int J Paediatr Dent.

[55] Zhang S, Liu J, Lo EC, Chu C-H (2014). Dental caries status of Bulang preschool children in Southwest China. BMC Oral Health.

[56] Tanaka K, Miyake Y (2014). Low birth weight, preterm birth or small-for-gestational-age are not associated with dental caries in young Japanese children. BMC Oral Health.

[57] Colombo S, Gallus S, Beretta M, Lugo A, Scaglioni S, Colombo P, Paglia M, Gatto R, Marzo G, Caruso S (2019). Prevalence and determinants of early childhood caries in Italy. Eur J Paediatr Dent.

[58] Agouropoulos A, Birpou E, Twetman S, Kavvadia K (2019). Validation of three caries risk assessment tools for preschool children from areas with high caries prevalence. Pediatr Dent.

[59] Musinguzi N, Kemoli AM, Okullo I (2019). Prevalence and treatment needs for early childhood caries among 3-5-year-old children from a rural community in Uganda. Front Public Health.

[60] Montes GR, Bonotto DV, Ferreira FM, Menezes JVNB, Fraiz FC. Caregiver’s oral health literacy is associated with prevalence of untreated dental caries in preschool children. Cien Saude Colet. 2019;24(7):2737-44. 10.1590/1413-81232018247.18752017 31340290

[61] Boustedt K, Dahlgren J, Twetman S, Roswall J. Tooth brushing habits and prevalence of early childhood caries: a prospective cohort study. Eur Arch Paediatric Dent. 2019:1–5. 10.1007/s40368-019-00463-3 31338770

[62] Tonpe M, Patil RU, Kadam A, Bayad P, Shetty V, Vinay V (2019). Comparative evaluation of two caries detection systems for detecting the prevalence of early childhood caries: a cross-sectional study. Dental Res J.

[63] Wang Z, Rong W, Zhang Y, Zeng X, Li Z, Liu Z (2019). Prevalence and contributing factors of dental caries of 6-year-old children in four regions of China. PeerJ.

[64] Nomura Y, Maung K, Khine K, Min E, Sint KM, Lin MP, Myint W, Khaing M, Aung T, Sogabe K (2019). Prevalence of dental caries in 5-and 6-year-old Myanmar children. Int J Dent.

[65] Wu X, Wang J, Cai T, Li Y, Zhou Z, Yang Z (2019). Prevalence and influencing factors of deciduous caries in preschool children in Chongqing city. Hua Xi Kou Qiang Yi Xue Za Zhi.

[66] Abbass MM, Mahmoud SA, El Moshy S, Rady D, AbuBakr N, Radwan IA, Ahmed A, Abdou A, Al Jawaldeh A (2019). The prevalence of dental caries among Egyptian children and adolescences and its association with age, socioeconomic status, dietary habits and other risk factors. A cross-sectional study. F1000Research.

[67] Goenka P, Dutta S, Marwah N, Sarawgi A, Nirwan M, Mishra P (2018). Prevalence of dental caries in children of age 5 to 13 years in district of Vaishali, Bihar, India. Int J Clin Pediatr Dent.

[68] Chugh VK, Sahu KK, Chugh A (2018). Prevalence and risk factors for dental caries among preschool children: a cross-sectional study in eastern India. Int J Clin Pediatr Dent.

[69] Vandana K, Raju SH, Badepalli RR, Narendrababu J, Reddy C, Sudhir K (2018). Prevalence and risk-factors of early childhood caries among 2–6-year-old Anganwadi children in Nellore district, Andhra Pradesh, India: a cross-sectional survey. Indian J Dent Res.

[70] Igic M, Obradovic R, Filipovic G (2018). Prevalence and progression of early childhood caries in Nis, Serbia. Eur J Paediatr Dent.

[71] Kato H, Tanaka K, Shimizu K, Nagata C, Furukawa S, Arakawa M, Miyake Y (2017). Parental occupations, educational levels, and income and prevalence of dental caries in 3-year-old Japanese children. Environ Health Prev Med.

[72] Li Y, Wulaerhan J, Liu Y, Abudureyimu A, Zhao J (2017). Prevalence of severe early childhood caries and associated socioeconomic and behavioral factors in Xinjiang, China: a cross-sectional study. BMC Oral Health.

[73] Mangla RG, Kapur R, Dhindsa A, Madan M (2017). Prevalence and associated risk factors of severe early childhood caries in 12-to 36-month-old children of Sirmaur district, Himachal Pradesh, India. Int J Clin Pediatr Dent.

[74] Owen M, Ghanim A, Elsby D, Manton D (2018). Hypomineralized second primary molars: prevalence, defect characteristics and relationship with dental caries in Melbourne preschool children. Aust Dent J.

[75] Pal A, Gupta S, Rao A, Kathal S, Roy S, Pandey S (2017). Family-related factors associated with caries prevalence in the primary dentition of 5–6-year-old children in urban and rural areas of Jabalpur City. Contemp Clin Dent.

[76] Wagne Y, Heinrich-Weltzien R (2017). Caries prevalence and risk assessment in Thuringian infants, Germany. Oral Health Prev Dent.

[77] Shah N, Mathur VP, Kant S, Gupta A, Kathuria V, Haldar P, Pandey RM (2017). Prevalence of dental caries and periodontal disease in a rural area of Faridabad District, Haryana, India. Indian J Dent Res.

[78] Yuan S, Shi L, Lv J (2017). Survey on dental caries prevalence among 3-year-old children in Jing’an district of Shanghai. Shanghai Kou Qiang Yi Xue.

[79] Jiang Y-Y, Jiang Y-Y (2017). Prevalence of early childhood caries among 2-to 5-year-old preschoolers in kindergartens of Weifang City, China: a cross-sectional study. Oral Health Prev Dent.

[80] Massignan C, Ximenes M, da Silva Pereira C, Dias L, Bolan M, Cardoso M (2016). Prevalence of enamel defects and association with dental caries in preschool children. Eur Arch Paediatr Dent.

[81] Koya S, Ravichandra K, Arunkumar VA, Sahana S, Pushpalatha H (2016). Prevalence of early childhood caries in children of west Godavari District, Andhra Pradesh, South India: an epidemiological study. Int J Clin Pediatr Dent.

[82] Mothupi KA, Nqcobo CB, Yengopal V (2016). Prevalence of early childhood caries among preschool children in Johannesburg, South Africa. J Dent Child.

[83] Alkhtib A, Ghanim A, Temple-Smith M, Messer LB, Pirotta M, Morgan M (2016). Prevalence of early childhood caries and enamel defects in four and five-year old Qatari preschool children. BMC Oral Health.

[84] Henry JA, Muthu MS, Saikia A, Asaithambi B, Swaminathan K (2017). Prevalence and pattern of early childhood caries in a rural south Indian population evaluated by ICDAS with suggestions for enhancement of ICDAS software tool. Int J Paediatr Dent.

[85] Šačić L, Marković N, Muratbegović AA, Zukanović A, Kobašlija S (2016). The prevalence and severity of early childhood caries in preschool children in the Federation of Bosnia and Herzegovina. Acta Med Acad.

[86] Al-Meedani LA, Al-Dlaigan YH (2016). Prevalence of dental caries and associated social risk factors among preschool children in Riyadh, Saudi Arabia. Pak J Med Sci.

[87] Elidrissi SM, Naidoo S (2016). Prevalence of dental caries and toothbrushing habits among preschool children in Khartoum state, Sudan. Int Dent J.

[88] Gopal S, Chandrappa V, Kadidal U, Rayala C, Vegesna M (2016). Prevalence and predictors of early childhood caries in 3-to 6-year-old south Indian children--a cross-sectional descriptive study. Oral Health Prev Dent.

[89] Aghighi S, Omrani L (2010). Evaluation of DMFT index and level of teeth and mouth hygiene education among students in air force military bases in the year 2008-2009. EBNESINA.

[90] Mortazavi M, Ebrahimi Z (1997). The prevalence of dental decay in 6 to 9 year old children in shiraz. J Dent.

[91] Asdagh S, Nuroloyuni S, Amani F, Sadeghi MT (2015). Dental caries prevalence among 6–12 years old school children in Ardabil city, 2012.

[92] Memar N, Ghazizade A, Mahmoudi SH (1999). DMFT index (decayed-missing-filled Teath) and its effective factors in 12-year-old students in Sanandaj. Kurdistan Med J.

[93] Javadine Jad SH, Karami M, Azizi HR (2006). Caries prevalence in 12-year -o ld children of I sfahan City expressed by the significant caries index. MUI..

[94] Sadeghi M (2007). Prevalence and bilateral occurrence of first permanent molar caries in 12-year-old students. J Dent Res Dent Clin Dental Prospects.

[95] Yousofi M, Behrouzpour K, Kazemi S, Afroughi S (2015). Dental caries and related factors among 7-12 year-old school children in Yasuj, Iran, in 2014. Armaghane Danesh.

[96] Eskandarizadeh A, Sajadi FS, Torabi M, Sharifi M, Amini Z, Sahebghalam B, Mahdavi SS, Asadpoor S, Ehsan N, Saeedi V (2015). Caries free prevalence among 6, 12 & 15-year old school children in Kerman during 2000-2005. J Health Dev.

[97] Mossaheb P, Abadi A, Amini M (2011). The relationship between food intake and dental caries in a group of Iranian children in 2009. Res Dent Sci.

[98] Qin D, Jiang HF, Shen L, Zhang C, Chai ZW, Wang JH (2019). Prevalence of dental caries and associated factors among 10-12-year-old students in Chongqing. Hua Xi Kou Qiang Yi Xue Za Zhi.

[99] Alshehhi A, Al Halabi M, Hussein I, Salami A, Hassan A, Kowash M (2020). Enamel defects and caries prevalence in preterm children aged 5-10 years in Dubai. Libyan J Med.

[100] Cheng Y-H, Liao Y, Chen D-Y, Wang Y, Wu Y (2019). Prevalence of dental caries and its association with body mass index among school-age children in Shenzhen, China. BMC Oral Health.

[101] Villanueva-Gutiérrez T, Irigoyen-Camacho ME, Castaño-Seiquier A, Zepeda-Zepeda MA, Sanchez-Pérez L, Frechero NM (2019). Prevalence and severity of molar–incisor hypomineralization, maternal education, and dental caries: a cross-sectional study of Mexican schoolchildren with low socioeconomic status. J Int Soc Prev Community Dent.

[102] Lešić S, Dukić W, Šapro KZ, Tomičić V, Kadić S (2019). Caries prevalence among schoolchildren in urban and rural Croatia. Cent Eur J Public Health.

[103] Mohd Nor NA, Chadwick B, Farnell D, Chestnutt I (2019). The prevalence of enamel and dentine caries lesions and their determinant factors among children living in fluoridated and non-fluoridated areas. Community Dent Health.

[104] Vanvitelli CL (2019). Prevalence of caries and dental malocclusions in the apulian paediatric population: an epidemiological study. Eur J Paediatr Dent.

[105] Obregón-Rodríguez N, Fernández-Riveiro P, Piñeiro-Lamas M, Smyth-Chamosa E, Montes-Martínez A, Suárez-Cunqueiro M (2019). Prevalence and caries-related risk factors in schoolchildren of 12-and 15-year-old: a cross-sectional study. BMC Oral Health.

[106] Mimoza C, Vito MA (2019). Evaluation of caries prevalence and decayed-, missing-, and filled-teeth values in permanent dentition in children 7 to 10 years old-a longitudinal study. J Contemp Dent Pract.

[107] Aldossary MS, Alamri AA, Alshiha SA, Hattan MA, Alfraih YK, Alwayli HM (2018). Prevalence of dental caries and fissure sealants in the first permanent molars among male children in Riyadh, Kingdom of Saudi Arabia. Int J Clin Pediatr Dent.

[108] Ballouk MA-H, Dashash M (2019). Caries prevalence and dental health of 8–12 year-old children in Damascus city in Syria during the Syrian crisis; a cross-sectional epidemiological oral health survey. BMC Oral Health.

[109] Alhabdan YA, Albeshr AG, Yenugadhati N, Jradi H (2018). Prevalence of dental caries and associated factors among primary school children: a population-based cross-sectional study in Riyadh, Saudi Arabia. Environ Health Prev Med.

[110] Konde S, Darshini CS, Agarwal M, Peethambar P (2018). Unrevealed caries in unerupted teeth: a prevalence study. Contemp Clin Dent.

[111] Solis-Riggioni A, Gallardo-Barquero C, Chavarria-Bolaños D (2018). Prevalence and severity of dental caries in foster-care children and adolescents. J Clin Pediatr Dent.

[112] Musa TH, Li W, Li X, Wang WX, Soro WL, Gao R, Song Y, He Y, Hong L, Musa HH (2018). Prevalence of dental caries profile in children and adolescents in rural Jiangsu Province. Arch Dis Child.

[113] Dutra ER, Chisini LA, Cademartori MG, Oliveira LJC d, Demarco FF, Correa MB (2018). Accuracy of partial protocol to assess prevalence and factors associated with dental caries in schoolchildren between 8-12 years of age. Cad Saude Publica.

[114] Al-Akwa AA, Al-Maweri SA (2018). Dental caries prevalence and its association with fluoride level in drinking water in Sana’a, Yemen. Eur J Dent.

[115] Cruz MGB d, Narvai PC (2018). Caries and fluoridated water in two Brazilian municipalities with low prevalence of the disease. Rev Saude Publica.

[116] Andegiorgish AK, Weldemariam BW, Kifle MM, Mebrahtu FG, Zewde HK, Tewelde MG, Hussen MA, Tsegay WK (2017). Prevalence of dental caries and associated factors among 12 years old students in Eritrea. BMC Oral Health.

[117] Alwayli HM, Alshiha SA, Alfraih YK, Hattan MA, Alamri AA, Aldossary MS (2017). A survey of fissure sealants and dental caries prevalence in the first permanent molars among primary school girls in Riyadh, Saudi Arabia. Eur J Dent.

[118] Dobbiani A, Berton F, Perinetti G, Costantinides F, Di RL (2018). Prevalence of dental caries among schoolchildren from north-eastern Italian population. Minerva Stomatol.

[119] Maran S, Shashikiran N, Ahirwar P, Maran P, Kannojiya PR, Niranjan B (2017). Prevalence of dental caries and traumatic dental injuries among 6-to 12-year-old children in Bhopal City, India. Int J Clini Pediatr Dent.

[120] Kim H-N, Kim J-H, Kim S-Y, Kim J-B (2017). Associations of community water fluoridation with caries prevalence and oral health inequality in children. Int J Environ Res Public Health.

[121] Sköld U (2016). Approximal caries increment in relation to baseline approximal caries prevalence among adolescents in Sweden with and without a school-based fluoride varnish programme. Community Dent Health.

[122] Plaka K, Ravindra K, Mor S, Gauba K (2017). Risk factors and prevalence of dental fluorosis and dental caries in school children of North India. Environ Monit Assess.

[123] Hiremath A, Murugaboopathy V, Ankola AV, Hebbal M, Mohandoss S, Pastay P (2016). Prevalence of dental caries among primary school children of India–a cross-sectional study. J Clin Diagn Res.

[124] Kottayi S, Bhat S, Hegde K, Peedikayil F, Chandru T, Anil S (2016). A cross-sectional study of the prevalence of dental caries among 12-to 15-year-old overweight schoolchildren. J Contemp Dent Pract.

[125] Ponnudurai Arangannal SKM, Jayaprakash J (2016). Prevalence of dental caries among school children in Chennai, based on ICDAS II. J Clin Diagn Res.

[126] Djossou D, Nancy J, Houinato D, Lanchoessi D (2015). Prevalence of dental caries in school in the city of Ouidah in 2013. Odonto-stomatol Trop.

[127] Weusmann J, Mahmoodi B, Azaripour A, Kordsmeyer K, Walter C, Willershausen B (2015). Epidemiological investigation of caries prevalence in first grade school children in Rhineland-Palatinate, Germany. Head Face Med.

[128] Goel R, Vedi A, Veeresha K-L, Sogi G-M, Gambhir R-S (2015). Oral hygiene practices and dental caries prevalence among 12 & 15 years school children in Ambala, Haryana-a cross-sectional study. J Clin Exp Dent.

[129] Farooqi FA, Khabeer A, Moheet IA, Khan SQ, Farooq I, ArRejaie AS (2015). Prevalence of dental caries in primary and permanent teeth and its relation with tooth brushing habits among schoolchildren in eastern Saudi Arabia. Saudi Med J.

[130] Arora G, Bhateja S (2015). Prevalence of dental caries, periodontitis, and oral hygiene status among 12-year-old schoolchildren having normal occlusion and malocclusion in Mathura city: a comparative epidemiological study. Indian J Dent Res.

[131] Sukhabogi JR, Parthasarathi P, Anjum S, Shekar B, Padma C, Rani A (2014). Dental fluorosis and dental caries prevalence among 12 and 15 year old school children in Nalgonda district, Andhra Pradesh, India. Ann Med Health Sci Res.

[132] Al-Darwish M, El Ansari W, Bener A (2014). Prevalence of dental caries among 12–14 year old children in Qatar. Saudi Dent J.

[133] Aidara A, Bourgeois D (2014). Prevalence of dental caries: national pilot study comparing the severity of decay (CAO) vs ICDAS index in Senegal. Odonto-stomatol Trop.

[134] Ingle NA, Dubey HV, Kaur N, Gupta R (2014). Prevalence of dental caries among school children of Bharatpur city, India. J Int Soc Prev Community Dent.

[135] Sofola O, Folayan M, Oginni A (2014). Changes in the prevalence of dental caries in primary school children in Lagos state, Nigeria. Niger J Clin Pract.

[136] Das D, Misra J, Mitra M, Bhattacharya B, Bagchi A (2013). Prevalence of dental caries and treatment needs in children in coastal areas of West Bengal. Contemp Clin Dent.

[137] Mahfouz M, Esaid AA (2014). Dental caries prevalence among 12–15 year old Palestinian children. Int Sch Res Notices.

[138] Riziwaguli A, Asiya Y, Liu Y, Yang R, Zou J (2013). Caries prevalence of the first permanent molar among 7-9 years old Uygur children in Urumqi, Xinjiang autonomous region. Shanghai Kou Qiang Yi Xue.

[139] Joshi N, Sujan S, Joshi K, Parekh H, Dave B (2013). Prevalence, severity and related factors of dental caries in school going children of Vadodara city–an epidemiological study. J Int Oral Health.

[140] Pieper K, Lange J, Jablonski-Momeni A, Schulte A (2013). Caries prevalence in 12-year-old children from Germany: results of the 2009 national survey. Community Dent Health.

[141] Yengopal V, Nqcobo C, Thekiso M, Rudolph M, Joosab Z (2012). Dental caries prevalence in children attending special needs schools in Johannesburg, Gauteng Province, South Africa. South Afr Dent J.

[142] Murthy A, Pramila M, Ranganath S (2014). Prevalence of clinical consequences of untreated dental caries and its relation to dental fear among 12–15-year-old schoolchildren in Bangalore city, India. Eur Arch Paediatr Dent.

[143] Koposova N, Eriksen HM, Widstrom E, Handegard B, Pastbin M, Koposov R (2013). Caries prevalence and determinants among 12-year-olds in north-West Russia and northern Norway. Stomatologija.

[144] Dixit A, Hao F, Mukherjee S, Lakshman T, Kompella R (2013). Towards an elastic distributed SDN controller. ACM SIGCOMM Comput Commun Rev.

[145] Suprabha BS, Rao A, Shenoy R, Khanal S (2013). Utility of knowledge, attitude, and practice survey, and prevalence of dental caries among 11-to 13-year-old children in an urban community in India. Glob Health Action.

[146] Panagidis D, Schulte A (2012). Caries prevalence in 12-year-old Cypriot children. Community Dent Health.

[147] Shailee F, Sogi G, Sharma K, Nidhi P (2012). Dental caries prevalence and treatment needs among 12-and 15-year old schoolchildren in Shimla city, Himachal Pradesh, India. Indian J Dent Res.

[148] Lagana G, Fabi F, Abazi Y, Kerçi A, Jokici M, Nastasi EB, Vinjolli F, Cozza P (2012). Caries prevalence in a 7-to 15-year-old Albanian schoolchildren population. Ann Stomatol.

[149] Subedi B, Shakya P, Kc U, Jnawali M, Paudyal B, Acharya A, Koirala S, Singh A (2011). Prevalence of dental caries in 5-6 years and 12-13 years age group of school children of Kathmandu valley. J Nepal Med Assoc.

[150] Oulis CJ, Berdouses ED, Mamai-Homata E, Polychronopoulou A (2011). Prevalence of sealants in relation to dental caries on the permanent molars of 12 and 15-year-old Greek adolescents. A national pathfinder survey. BMC Public Health.

[151] Jamelli SR, Rodrigues CS, de Lira PIC (2010). Nutritional status and prevalence of dental caries among 12-year-old children at public schools: a case-control study. Oral Health Prev Dent.

[152] Kanagaratnam S, Schluter P, Durward C, Mahood R, Mackay T (2009). Enamel defects and dental caries in 9‐year‐old children living in fluoridated and nonfluoridated areas of Auckland, New Zealand. Community Dent Oral Epidemiol.

[153] Bissar A-R, Schulte AG, Muhjazi G, Koch MJ (2007). Caries prevalence in 11-to 14-year old migrant children in Germany. Int J Public Health.

[154] Ferro R, Besostri A, Meneghetti B, Stellini E (2007). Prevalence and severity of dental caries in 5-and 12-year old children in the Veneto region (Italy). Community Dent Health.

[155] Moreira PVL, Rosenblatt A, Severo AMR (2006). Prevalence of dental caries in obese and normal-weight Brazilian adolescents attending state and private schools. Community Dent Health.

[156] Schulte AG, Momeni A, Pieper K (2006). Caries prevalence in 12-year-old children from Germany. Results of the 2004 national survey. Community Dent Health.

[157] Paredes VG, Paredes CC, Mir BP (2006). Prevalence of dental caries: comparison between immigrant and autochthonous children. An Pediatr (Barc).

[158] Mestriner SF, Pardini LC, Mestriner WJ (2006). Impact of the bitewing radiography exam inclusion on the prevalence of dental caries in 12-year-old students in the city of Franca, São Paulo, Brazil. J Appl Oral Sci.

[159] Traebert J, Suárez CS, Onofri DA, Marcenes W (2002). Prevalence and severity of dental caries and treatment needs in small Brazilian counties. Cad Saude Publica.

[160] Nag R, Bihani VK, Panwar VR, Acharya J, Bihani T, Pandey R (2012). Prevalence of dental caries and treatment needs in the school going children in Bikaner, Rajasthan-an observational study. J Indian Dent Assoc.

[161] Kumar S, Tadakamadla J, Kroon J, Johnson NW (2016). Impact of parent-related factors on dental caries in the permanent dentition of 6-12-year-old children: a systematic review. J Dent.

[162] Hermes Soares G, Fernanda Pereira N, Gabriela Haye Biazevic M, Minatel Braga M, Michel-Crosato E (2019). Dental caries in south American indigenous peoples: a systematic review. Community Dent Oral Epidemiol.

[163] Marghalani AA, Guinto E, Phan M, Dhar V, Tinanoff N (2017). Effectiveness of xylitol in reducing dental caries in children. Pediatr Dent.

[164] Hegde MN, Attavar SH, Shetty N, Hegde ND, Hegde NN (2019). Saliva as a biomarker for dental caries: a systematic review. J Conserv Dent.

[165] Masaheb P, Kargarnovin Z, Malekafzali B, Abadi AR, Amini M (2011). The relationship between food intake and dental caries in a group of Iranian children in 2009. J Res Dent Sci.

[166] Prakash P, Subramaniam P, Durgesh BH, Konde S (2012). Prevalence of early childhood cariesand associated risk factors in preschool children of urban Bangalore, India: a cross-sectional study. Eur J Dent.

[167] Namal N, Yuceokur AA, Can G (2009). Significant caries index values and related factors in 5-6-year-old children in Istanbul, Turkey. East Mediterr Health J.

[168] Wagner Y, Heinrich-Weltzien R (2017). Caries prevalence and risk assessment in Thuringian infants, Germany. Oral Health Prev Dent.

